# Informed sequential pooling approach to detect SARS-CoV-2 infection

**DOI:** 10.1371/journal.pone.0244475

**Published:** 2020-12-30

**Authors:** Renato Millioni, Cinzia Mortarino

**Affiliations:** 1 Fascial Manipulation Institute by Stecco, Padova, Italy; 2 Department of Statistical Sciences, University of Padova, Padova, Italy; Universita degli Studi di Parma, ITALY

## Abstract

The alarming spread of the pandemic coronavirus disease 2019 (COVID-19) caused by the SARS-CoV-2 virus requires several measures to reduce the risk of contagion. Every successful strategy in controlling the SARS-CoV-2 infection depends on timely diagnosis, which should include testing of asymptomatic carriers. Consequently, increasing the throughput for clinical laboratories for the purposes of conducting large-scale diagnostic testing is urgently needed. Here we support the hypothesis that standard diagnostic protocol for SARS-CoV-2 virus could be conveniently applied to pooled samples obtained from different subjects. We suggest that a two-step sequential pooling procedure could identify positive subjects, ensuring at the same time significant benefits of cost and time. The simulation data presented herein were used to assess the efficiency, in terms of number of required tests, both for random assignment of the subjects to the pools and for situations in which epidemiological and clinical data are used to create "informed" pools. Different scenarios were simulated to measure the effect of different pool sizes and different values for virus frequency. Our results allow for a customization of the pooling strategy according to the specific characteristics of the cohort being tested.

## Introduction

The pandemic coronavirus disease 2019 (COVID-19) is caused by the SARS-CoV-2 virus. The infection is predominantly transmitted through large droplets and by contact with infected surfaces or fomites. The alarming spread of the infection and the severe clinical disease that it may cause have led to the implementation of several measures to reduce the risk of contagion. Active case detection, rapid case isolation, and contact quarantine, as well as rigorous application of infection control practices are successful strategies in controlling SARS-CoV-2 infection outbreaks. The success of these strategies relies initially on viral diagnosis. The overloading to which the laboratories are currently subjected causes a cascade delay of all virus containment procedures with potentially dramatic results for prevention of the infection.

In most countries, testing for COVID-19 is mainly restricted to people with symptoms. However, a large percentage of asymptomatic subjects is estimated to exist [1]. Asymptomatic spread has likely driven the silent growth of the SARS-CoV-2 epidemic, which emerged only when health systems began to collapse. Asymptomatic cases play a significant role in infection transmission, also considering that the chance of transmission through inanimate surfaces is less frequent than previously recognised [2]. It is therefore essential that the degree to which asymptomatic individuals affect viral diffusion be evaluated [3]. Tracing contacts of known positive cases, travel bans, and social distancing are the main strategies for reducing the risk of contagion by asymptomatic subjects. A widespread testing strategy to screen asymptomatic subjects could be useful in reducing transmission of SARS-CoV-2, but this approach is highly challenging taking into account of the amount of work, time, and cost that it would entail.

For this reason, we propose here a pre-screening strategy which should increase the capacity for clinical laboratories to conduct large-scale diagnostic testing, enough to screen a significant portion of the asymptomatic population.

SARS-CoV-2 is an enveloped virus containing a single strand of positive-sense RNA, and its diagnostic protocol is a RT-PCR assay, as previously described ([4,5]). Briefly, SARS-CoV-2 has been detected from a variety of upper and lower respiratory sources including throat, nasal nasopharyngeal (NP), sputum, and bronchial fluid ([6,7]). Oropharyngeal (OP) and NP swabs are the most frequently used samples. The sampling is carried out using two distinct swabs which can be inserted in the same test tube containing the viral transport medium to increase the yield for RT-PCR analysis [8]. Recent studies have shown that the SARS-CoV-2 detection can also be correctly applied on saliva, with the advantage of an easier sample collection [9]. Total RNA is extracted and SARS-CoV-2 target genes are simultaneously amplified and tested during the quantitative RT-PCR assay.

Recently, Hogan et al. (2020) [10] performed a retrospective study on SARS-CoV-2 based on sample pooling. The roots of this idea go back to Dorfman [11]. “Pooling” means that swab samples taken from different subjects can be combined before the RNA extraction phase. These authors used 2888 samples from nasopharyngeal and bronchoalveolar lavages that were collected between January 1, 2020, and February 26, 2020, from subjects who had not been tested for SARS-CoV-2. Nine or ten samples were pooled, and screening was performed by RT-PCR. A total of 292 pools were screened and the confirmed positivity rate for SARS-CoV-2 was 0.07% (2/2888). The aim of pooling is to reduce the number of test kits used, significantly shortening the time and costs of analysis.

A single positive sample can be properly detected in pools of up to 32 or 64 samples, using standard kits and protocols, with an eventual slight increase in the PCR cycle threshold [12]. Some pre-prints claim that it is also technically possible to detect a single positive sample in largerpool sizes, with samples of up to 100, 120, or even 1000 [13]. However, the possibility of a single positive sample escaping detection in such large pools, especially if the viral load is low, must be taken into account. This could happen especially for samples at the initial or final phase of infection, regardless of whether the patient is symptomatic or not [14]. Various proposals have already been made to reduce the risk of increase false negative results due to pooling such as incrementing the capability of the extraction protocol [15]. Moreover, it must be considered that a small reduction in sensitivity should be conveniently balanced by the possibility of screening more people more often. By significantly reducing the number of analysis, pooling offers the possibility of increasing the frequency of monitoring, which is probably the most important factor to achieve an effective surveillance strategy [16].

However, the suitability of pool size does not depend only on the limit of sensitivity of the RT-PCR, but must also be set on the basis of statistical evaluations that are the subject of this publication.

The basic concepts for understanding the pooling strategy are simple: 1) only a pool made up of all negative samples will give a negative result for the pool analysis; and 2) a single positive sample within a pool makes the result of the pool analysis positive. If the pool is positive, it is necessary to proceed to individual testing for the purposes of identifying true positives (TP) and false positives (FP; i.e., a negative subject whose swab has been mixed with at least one positive swab). As all individual samples in a negative pool are considered as true negative (TN), the pooling approach significantly reduces time and cost when a large proportion of pools tests negative. However, it is clear that the effectiveness of pooling is inversely proportional to the frequency of the virus in the selected cohort and, as we will demonstrate more precisely in the results section, this approach can be inefficient or even counter-productive if the presence of the virus is high.

The aim of this paper is to i) propose a two-step sequential pooling strategy; ii) identify the variables for which the pooling method can be more or less effective; and iii) to develop strategies to further improve this approach. We began by identifying the main variables to be included in our model. The first and perhaps most important variable, as already mentioned, is the frequency of the virus. Unfortunately, this information is not known *a priori*, but can be estimated. The second variable is the effectiveness of the clinical and epidemiological criteria that are adopted to create the pools, compared to an analysis in which these pools are created randomly. The third variable is the size of the pool. We have taken into consideration a wide range of scenarios to adjust the variables to give the best results using fewer tests.

As certain strategies have the potential to improve the pooling approach, we compared alternative methods of pool creation and evaluated their different performance in relation to the variables described above. Our data suggest that a pre-screening strategy based on the use of a sequential informed pooling approach ensures that, in the most favourable conditions with low virus frequency, the number of required tests can drop to 20% of those required for individual testing. Higher virus frequencies still make sequential pooling efficient, provided that pool size is decreased and/or reliable epidemiological and clinical data are used for pool creation.

## Methods

The volume of samples initially collected from an individual must be enough for both pooled and individual follow-up testing. Alternatively, subjects requiring validation will be subjected to a new swab. This may be the most convenient choice only for very low viral frequency, as few repetitions of the tests would be expected. No patients were recruited specifically for this study.

Sequential pooling workflow follows these steps:

1. Assume that samples are arranged on a grid. A portion of each sample is collected to create a homogeneous pool following each row: "horizontal" pooling (pool H).
2. Perform the RT-PRC analysis of the “H” pools, each of size *s*. Based on these results, all negative pools can be excluded from further investigation, as they solely contain samples from TN subjects. Should all pools test negative, the procedure is complete.
3. Using samples not excluded in step B, create the vertical pools (pool V) following each column of the grid and perform RT-PCR analysis of the V pools (vertical pooling). The V pools will have the same size *s*, but their composition will be different from that of H pools, even if step B did not exclude any pool. Again, all negative pools can be excluded from further investigation, as they only contain samples from TN subjects.
4. Validation phase: Search for true positives (TP) and false positives (FP) by performing the RT-PCR analysis (*deconvolution* process).

Informed sequential pooling follows the same procedure as Sequential pooling, with the only difference being that a score for the probability of being infected will be associated to each subject in order to tag the subject as “suspected positive” or “suspected negative.” The standard diagnostic protocol has so far been applied in an emergency situation for which priority has been given especially to those who manifested symptoms. In this model, however, independence among samples is assumed, and other possible correlations are neglected (e.g. family members or co-workers should preferably be pooled together). The aim is to include in the same pool subjects with higher scores, avoiding their random spreading in the matrix. The correct assignment of this score would be accomplished by compiling a dedicated online questionnaire consisting of a few multiple choice questions. Results would be processed automatically, without being time consuming. The score is calculated on the basis of clinical and epidemiological criteria that have already been associated with a higher risk of acquiring COVID-19 [17]. For instance, susceptibility seems to be strongly associated with age and biological sex ([18–20]) suggesting that these simple criteria may play an important role in pool assignment.

**Fig 1:**
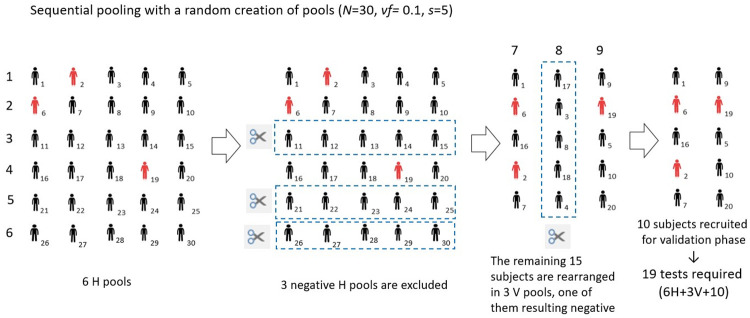
Graphic representation of the sequential pooling approach. The cohort dimension is *N* = 30, the pool size is 5, and the virus frequency, *vf*, is 0.10. To begin, the 30 samples are used to create 6 horizontal (H) pools. Since 3 pools’ results are negative (where red icons represent positive subjects), we can exclude 15 subjects. The remaining 15 are used to create 3 vertical (V) pools. As one of these pools yields negative result, only 10 subjects require individual testing. In the end, the total number of tests is equal to 19 (9 pools and 10 validation tests).

**Fig 2:**
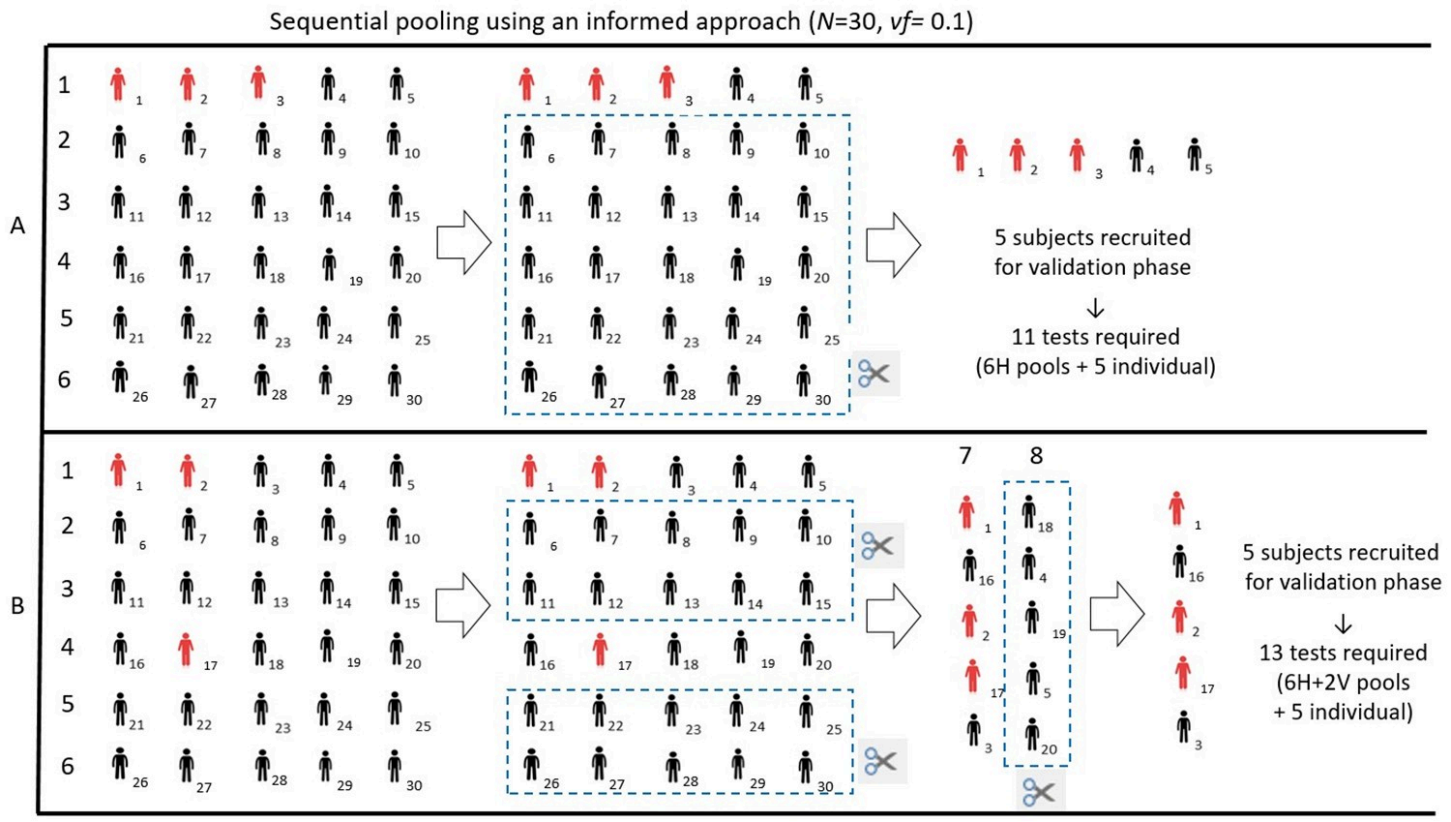
Graphic representation of the Informed sequential pooling approach. The cohort dimension is *N* = 30, the pool size is 5, and the *vf* is 0.10. Panels A and B show two possible scenarios based on available information allowing for the classification of subjects as either “suspect positive” or “suspect negative.” In scenario A, information allows for a concentration of all the positive subjects in the same horizontal pool. Thus after 6H pools, only 5 subjects are left, and vertical pooling is not necessary (globally 11 tests are required). In scenario B, information allows for a concentration of some of the positive samples but not all of them. Among 6 horizontal pools, 4 turn out to be negative. The 10 remaining subjects are then rearranged in 2 vertical pools. Since one pool gives a positive result, 5 validation tests are required (globally 13 tests).

Figs 1 and 2 show a simple graphic representation of the sequential pooling and informed sequential pooling approach, respectively. For the purposes of facilitating visual representation, we have chosen a test cohort of dimension *N* equal to 30.

In Fig 2, the upper panel shows a hypothetical scenario for which all positive subjects are grouped in the first pool. This result can be obtained if the information available to classify subjects as “suspect positive” or “suspect negative” is optimal. In the lower panel, we show another scenario for which clinical and epidemiological information allowed a grouping of the positive subjects, which is only partially correct. However, in this case, the informed approach is still useful for improving the efficiency of the method compared to random pool creation.

## Results

In order to assess the advantage of this two-step sequential pooling strategy in comparison with a standard approach in which each subject’ swab is tested separately, we performed simulations under different conditions. Results were obtained with Wolfram Mathematica 12.1. The simulated analysis was based on an assumed group of *N* = 600 subjects. The size *s* of each pool (both H and V) was allowed to vary from 2 to 300. The list of possible sizes *s* to split *N* = 600 subjects is equal to {2,3,4,5,6,8,10,12,15,20,24,25,30,40,50,60,75,100,120, 150,200,300}. We examined a virus frequency, *vf*, ranging from 0.01 to 0.30 (the latter situation thus corresponding to 30% of the subjects TP to the virus).

As a first step, we examined the performance of this strategy without using prior information about the subjects, that is, by creating pools completely at random. To do this, after setting *s* and *vf*, we performed 5,000 simulations and recorded the ratio between the total number of swab tests required, *T*, and *N*. For two-step sequential pooling, *T* includes both H and V pools required in steps B and C, but also validation tests in step D required on all the swabs from subjects not previously excluded. Since without a pooling strategy, *N* tests must be performed, the ratio *T/N* measures the efficacy of the proposed procedure. The smaller the value of this ratio, the fewer the number of required tests. Conversely, values close to 1 (or even above 1) would represent a useless (or a counter-productive) strategy.

**Fig 3:**
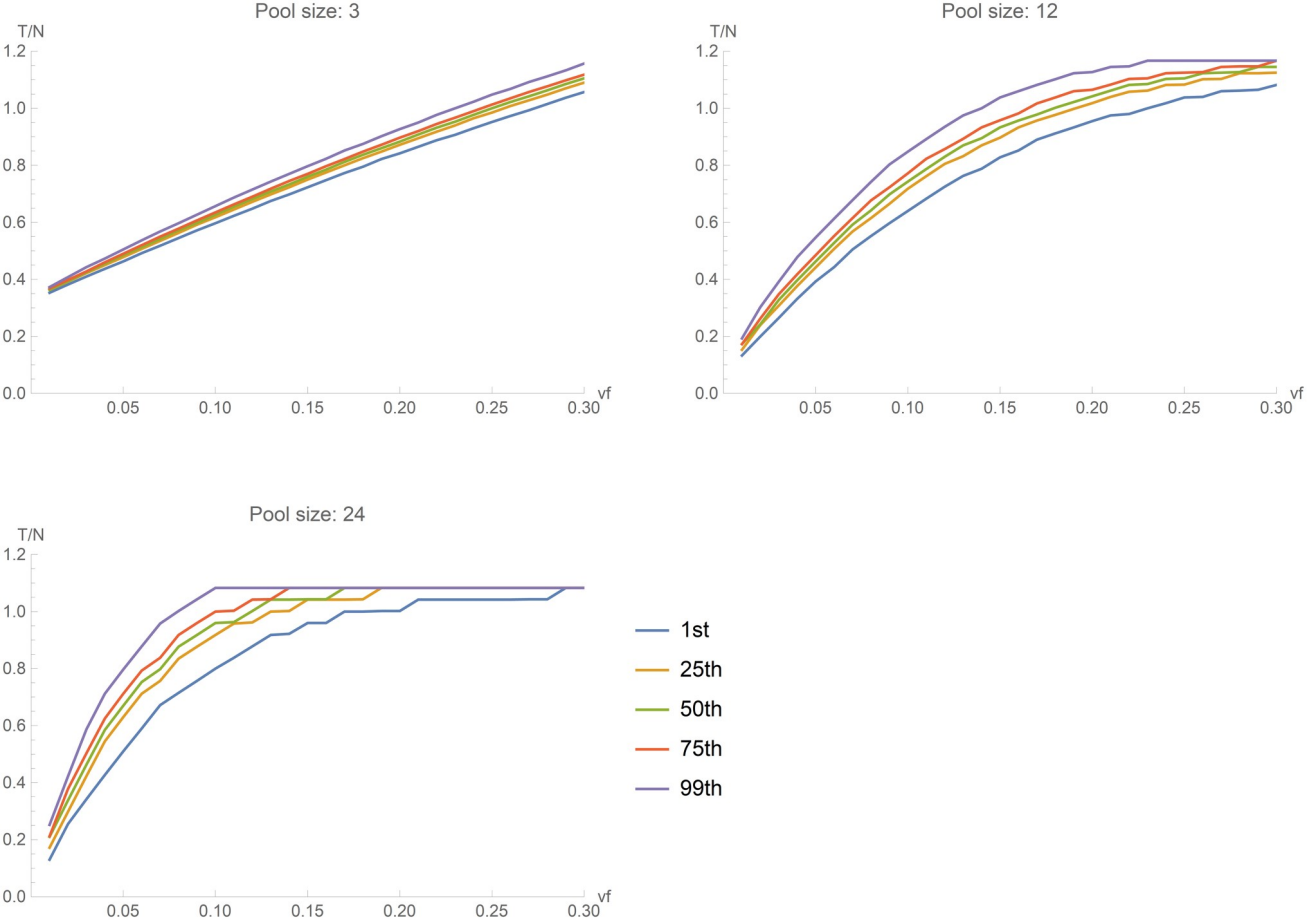
Random sequential pooling. The curves plotted represent the 1st, 25th, 50th (median), 75th, and 99th percentiles of *T/N* obtained in the set of 5,000 simulations, for three values of the pool size (*s* = 3,12,24).

Table 1 and Fig 3 show the results for *s* equal to 5, 12, and 24 (the entire set of plots is available in the S1 Fig). The curves plotted represent the 1st, 25th, 50th (median), 75th, and 99th percentiles of *T/N* obtained in the set of 5,000 simulations, for different *vf* values. In particular, given that the actual number of required tests depends on the random assignment of samples to pools, the 1st and 99th percentiles give an idea of the range of *T/N* between "favorable" or "unfavorable" assignments to the pools. The spread between the 25th and 75th, which is always very small in Fig 3, represents the central half of the simulations (after excluding the 25% more "favorable" and the 25% more "unfavorable" ones). As the pool size increases, we notice that the curves are less linear and the spread between the 1st and 99th percentile increases. For very small pools (*s* = 3) with a low virus frequency, the number of tests required in this approach is about 40% of the number of tests required separately testing each subject. As the value of *vf* increases, the number of tests grows slowly and pooling remains efficient (*T/N*<1) even if 25% of the subjects are positive in the group. Conversely, if we use larger pools (*s* = 24), the number of tests could drop to 20% for low virus frequency. However, the number of tests would increase faster as *vf* grows, and the procedure would be efficient only up to about 10% of positive subjects in the analysed cohort. In summary, the linear path of small pools ensures efficiency even for larger *vf*, but the nonlinear path observed for larger pools make them efficient for populations with a low virus presence.

**Table 1:** Random sequential pooling. Values of the 1st, 25th, 50th (median), 75th, and 99th percentiles of *T/N* obtained in the set of 5,000 simulations, for three values of the pool size (*s* = 3, 12, 24) and some values of the virus frequency (*vf* = 0.01, 0.10, 0.15, 0.20, 0.25, 0.30).

*T/N*	*vf*	1st	25th	50th	75th	99th
*s* = 3	0.01	0.353	0.363	0.363	0.368	0.373
	0.05	0.463	0.478	0.485	0.490	0.505
	0.10	0.597	0.618	0.627	0.635	0.657
	0.15	0.723	0.750	0.760	0.770	0.797
	0.20	0.842	0.872	0.883	0.897	0.927
	0.25	0.952	0.985	1.000	1.013	1.048
	0.30	1.057	1.090	1.105	1.118	1.157
*s* = 12	0.01	0.133	0.153	0.173	0.173	0.193
	0.05	0.393	0.442	0.463	0.485	0.547
	0.10	0.640	0.718	0.743	0.772	0.848
	0.15	0.828	0.897	0.933	0.958	1.038
	0.20	0.955	1.018	1.042	1.065	1.127
	0.25	1.038	1.083	1.105	1.125	1.167
	0.30	1.082	1.125	1.145	1.167	1.167
*s* = 24	0.01	0.130	0.172	0.210	0.212	0.252
	0.05	0.510	0.630	0.670	0.712	0.797
	0.10	0.800	0.918	0.960	1.000	1.083
	0.15	0.960	1.042	1.043	1.083	1.083
	0.20	1.002	1.083	1.083	1.083	1.083
	0.25	1.042	1.083	1.083	1.083	1.083
	0.30	1.083	1.083	1.083	1.083	1.083

**Fig 4:**
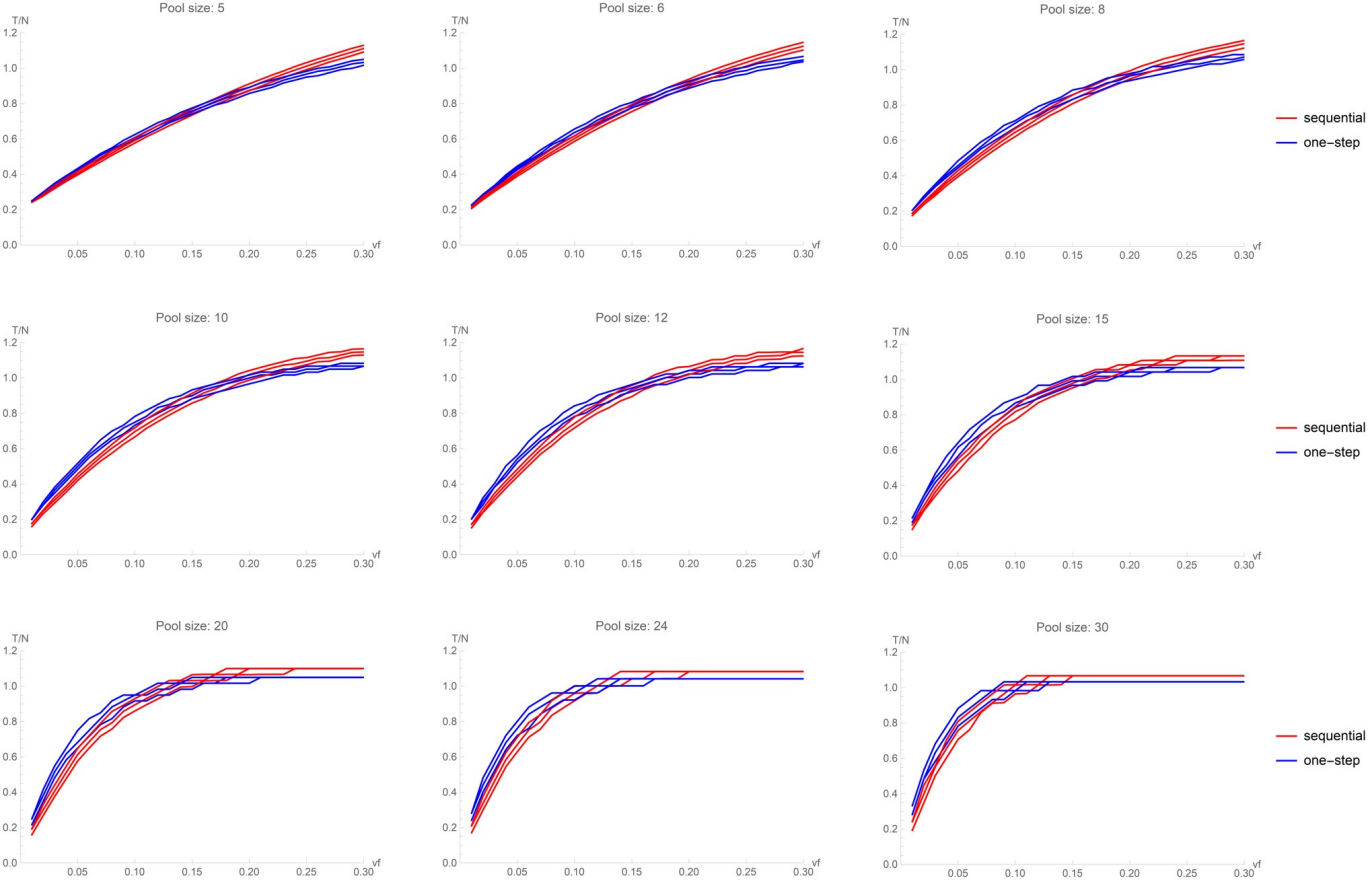
Random sequential pooling vs one-step pooling. The curves plotted represent the 25th, 50th (median), and 75th percentiles of *T/N* obtained in the set of 5,000 simulations, for 9 values of the pool size (*s* = 5, 6, 8, 10, 12, 15, 20, 24, 30).

As mentioned in the introduction, simple pooling was recently proposed for SARS-CoV-2 detection by Hogan et al. [10]. We notice that their study does not provide general efficiency results apart from their specific application, where pools of sizes 9 and 10 have been used and a very small *vf* has been reported (their value is even smaller than the smallest virus frequency assessed in our simulations). Their pooling strategy was originally proposed by Dorfman [11] and it is characterized by a pooling step followed by the validation phase. Conversely, the approach here proposed adds to the Dorfman scheme a further step, since two pooling steps have to performed before the validation phase. This is an important point to be considered when planning a pooling strategy, because this further step requires more time and organizational complexity within the laboratory. It is thus important to assess whether and under which conditions this increase in time and complexity generates an improvement in terms of efficiency. Fig 4 shows a comparison of a simple one-step pooling strategy with our two-step sequential procedure for different *vf* and *s* values. In this picture, the 25th, 50th (median), and 75th percentiles of *T/N* are shown. For very small pools (*s* = 5), they are almost equivalent. But, as soon as *s* is slightly increased to sensible values (ranging from 8 to 20), the sequential two-step pooling shows a better performance up to *vf* = 0.15. For bigger pools (*s* = 24, 30), we observe the same result up to *vf* around 0.10. For higher *vf*, both pooling strategies are counter-productive, as highlighted above for sequential pooling.

All of the previous results have been obtained assuming a completely random assignment of subjects to the pools. Often, however, clinical and epidemiological data about the subjects are available. If we could use these data to concentrate a portion of the positive subjects in the same horizontal pools, we would increase efficiency due to a higher number of negative pools at step B. In order to assess the savings of such an "informed pooling creation," we extended our simulations to different settings. We may conceive a scenario in which, prior to the test we detect a certain number of subjects, say *x*, that we expect to be positive (according to epidemiological criteria). We create *x/s* horizontal pools, each of size s, with those subjects. The remaining *(N-x)* subjects are assigned to the remaining *(N-x)/s* horizontal pools. Should epidemiological criteria be perfect, all the *x* subjects turn out to be true positive and thus the first *x/s* pools are positive. At the same time, all the *(N-x)* subjects without prior indication of an infection, with perfect epidemiological criteria, would be true negative and thus their *(N-x)/s* horizontal pools would yield a negative result. Of course, such an assumption is unrealistic and we expect that some of the *x* subjects suspected to be positive are true negative and also that some of the *(N-x)* subjects suspected to be negative are true positive.

Let us denote by α the fraction of the *vf∙N* true positive subjects in the population that are correctly assigned to the initial pools. The remaining (1-α) fraction is undetected and it is wrongly assigned to the second part of the pools. Criteria with perfect performance in prior detection of positive subjects would result in α = 1. In addition, let us denote by β the fraction of the (1-*vf)∙N* true negative subjects in the population that are correctly assigned to the final pools. The remaining (1-β) fraction is wrongly assigned to the first part of the pools. Criteria with perfect performance in prior detection of negative subjects would result in β = 1. For the same settings analysed in the random creation of the pools (*N* = 600, *vf* from 0.01 to 0.30, and *s* from 2 to 300), we explored the performance of the sequential procedure for different values of α and β. In particular, we allowed α and β to vary in the set {0.5, 0.6, 0.7, 0.8}. When both α and β are equal to 0.5, criteria are essentially unreliable and our situation is equivalent to the random assignment setting discussed above.

**Fig 5:**
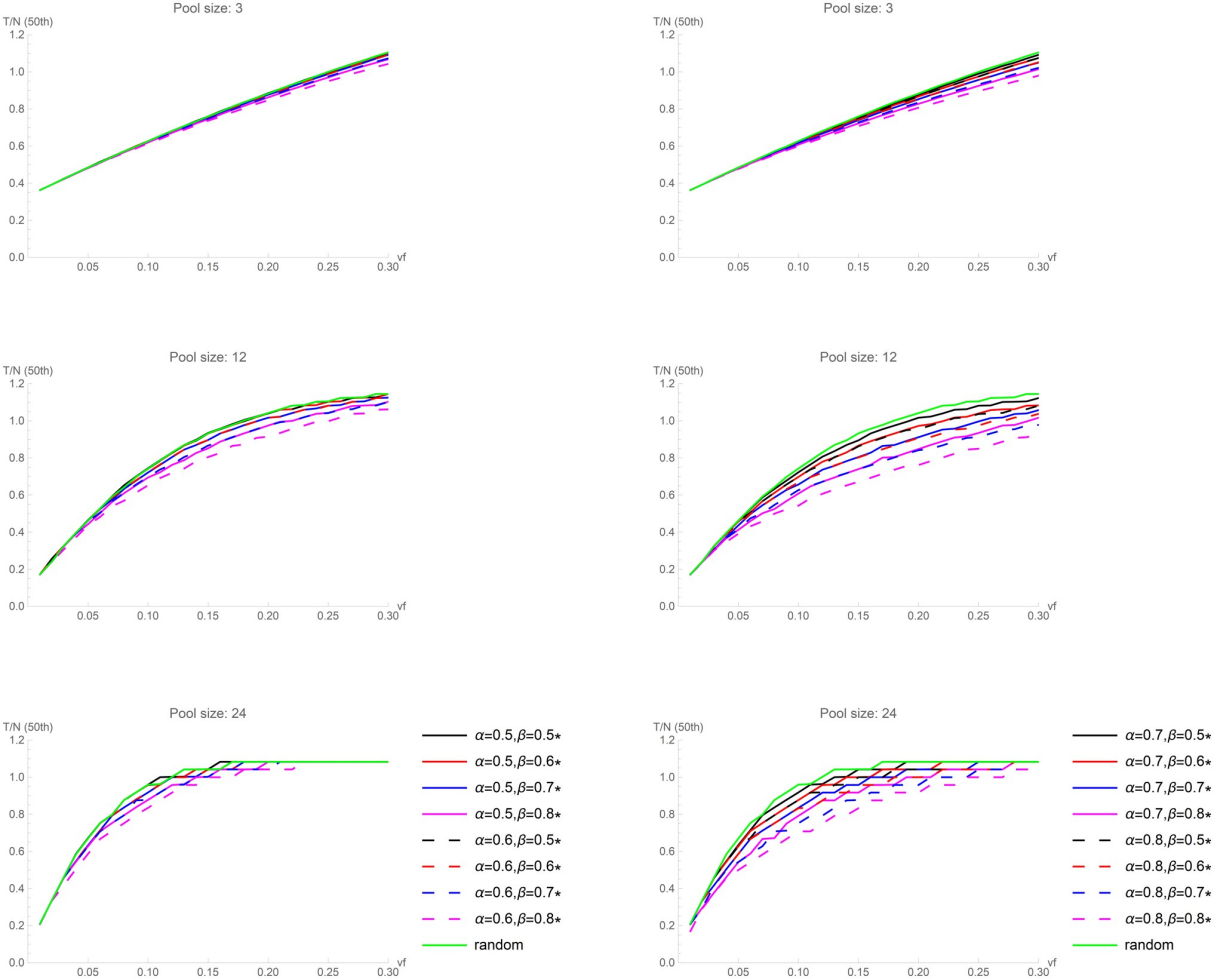
Informed sequential pooling. The curves plotted represent the 50th percentile (median) of *T/N* obtained in the set of 5,000 simulations, for three values of the pool size (*s* = 3,12,24). The upper plots were obtained with α = 0.5 and α = 0.6, combined with β = 0.5, 0.6, 0.7, 0.8. The lower plots were obtained with α = 0.7 and α = 0.8, combined with β = 0.5, 0.6, 0.7, 0.8.

Fig 5 shows the results of the simulations obtained for three values of the pool size (*s* = 3,12,24) for different combinations of α and β (the plots for the remaining *s* values are shown in the S2 and S3 Figs). Since there are many possible scenarios, to improve clarity we plotted only the 50th percentile (the values for all the 5 percentiles are displayed in Tables 2–4). Our aim is to compare the results of the number of tests required when swabs are randomly assigned to the pools with the number of tests required for different α and β values.

**Table 2:** Informed sequential pooling, pool size *s* = 3. Values of the 1st, 25th, 50th (median), 75th, and 99th percentiles of *T/N* obtained in the set of 5,000 simulations, for all combinations of α and β in {0.5, 0.6, 0.7, 0.8}, for some values of the virus frequency (*vf* = 0.01, 0.10, 0.15, 0.20, 0.25, 0.30).

		β = 0.5					β = 0.6					β = 0.7					β = 0.8				
		1st	25th	50th	75th	99th	1st	25th	50th	75th	99th	1st	25th	50th	75th	99th	1st	25th	50th	75th	99th
α = 0.5	*vf* = 0.01	0.353	0.363	0.363	0.368	0.373	0.353	0.363	0.363	0.368	0.373	0.357	0.363	0.363	0.368	0.373	0.353	0.363	0.363	0.368	0.373
	*vf* = 0.05	0.463	0.478	0.485	0.490	0.505	0.465	0.478	0.485	0.490	0.503	0.463	0.478	0.485	0.490	0.507	0.463	0.477	0.482	0.488	0.503
	*vf* = 0.10	0.597	0.618	0.627	0.635	0.657	0.597	0.618	0.627	0.635	0.657	0.595	0.615	0.625	0.633	0.653	0.590	0.612	0.620	0.628	0.650
	*vf* = 0.15	0.725	0.75	0.760	0.772	0.797	0.723	0.748	0.758	0.768	0.797	0.718	0.745	0.755	0.767	0.793	0.712	0.737	0.745	0.757	0.782
	*vf* = 0.20	0.842	0.872	0.885	0.897	0.927	0.840	0.870	0.883	0.895	0.925	0.833	0.865	0.877	0.888	0.920	0.822	0.850	0.863	0.875	0.903
	*vf* = 0.25	0.953	0.985	1.000	1.013	1.047	0.950	0.983	0.997	1.010	1.043	0.942	0.975	0.988	1.002	1.033	0.928	0.957	0.970	0.982	1.015
	*vf* = 0.30	1.055	1.090	1.105	1.120	1.155	1.053	1.087	1.102	1.117	1.152	1.045	1.077	1.092	1.105	1.138	1.025	1.055	1.068	1.082	1.115
α = 0.6	*vf* = 0.01	0.353	0.363	0.363	0.368	0.373	0.357	0.363	0.363	0.368	0.373	0.353	0.363	0.363	0.368	0.373	0.353	0.363	0.363	0.368	0.373
	*vf* = 0.05	0.463	0.478	0.485	0.490	0.507	0.463	0.478	0.485	0.490	0.503	0.463	0.477	0.483	0.488	0.503	0.462	0.475	0.482	0.487	0.502
	*vf* = 0.10	0.597	0.618	0.627	0.635	0.657	0.595	0.617	0.625	0.633	0.653	0.592	0.613	0.622	0.630	0.650	0.587	0.607	0.615	0.623	0.643
	*vf* = 0.15	0.723	0.748	0.760	0.770	0.797	0.718	0.745	0.755	0.767	0.792	0.712	0.738	0.748	0.758	0.785	0.702	0.727	0.737	0.747	0.772
	*vf* = 0.20	0.842	0.872	0.883	0.895	0.923	0.837	0.865	0.877	0.888	0.920	0.827	0.855	0.867	0.878	0.908	0.810	0.837	0.847	0.858	0.887
	*vf* = 0.25	0.952	0.983	0.997	1.010	1.047	0.943	0.975	0.988	1.002	1.035	0.932	0.962	0.975	0.987	1.018	0.908	0.938	0.950	0.962	0.992
	*vf* = 0.30	1.053	1.088	1.103	1.117	1.152	1.045	1.077	1.092	1.107	1.143	1.028	1.060	1.073	1.087	1.122	1.002	1.032	1.043	1.057	1.088
α = 0.7	*vf* = 0.01	0.353	0.363	0.363	0.368	0.373	0.353	0.363	0.363	0.368	0.373	0.353	0.363	0.363	0.368	0.373	0.353	0.363	0.363	0.368	0.373
	*vf* = 0.05	0.463	0.478	0.483	0.490	0.505	0.463	0.477	0.483	0.488	0.503	0.462	0.475	0.482	0.487	0.502	0.458	0.473	0.480	0.485	0.500
	*vf* = 0.10	0.595	0.617	0.625	0.633	0.653	0.593	0.613	0.622	0.630	0.652	0.587	0.608	0.617	0.625	0.647	0.578	0.600	0.608	0.617	0.637
	*vf* = 0.15	0.718	0.745	0.755	0.767	0.792	0.713	0.740	0.750	0.760	0.787	0.705	0.730	0.740	0.750	0.775	0.690	0.713	0.723	0.733	0.757
	*vf* = 0.20	0.835	0.865	0.877	0.890	0.918	0.827	0.855	0.868	0.880	0.910	0.813	0.842	0.853	0.865	0.893	0.790	0.818	0.828	0.838	0.865
	*vf* = 0.25	0.943	0.975	0.990	1.002	1.035	0.932	0.963	0.975	0.988	1.022	0.915	0.943	0.957	0.968	0.998	0.887	0.913	0.925	0.935	0.965
	*vf* = 0.30	1.042	1.077	1.092	1.105	1.142	1.030	1.062	1.075	1.088	1.122	1.007	1.038	1.050	1.063	1.093	0.975	1.003	1.015	1.027	1.053
α = 0.8	*vf* = 0.01	0.353	0.363	0.363	0.368	0.373	0.353	0.363	0.363	0.368	0.373	0.353	0.363	0.363	0.368	0.373	0.353	0.362	0.363	0.368	0.373
	*vf* = 0.05	0.463	0.477	0.483	0.490	0.503	0.462	0.477	0.482	0.488	0.503	0.458	0.475	0.480	0.487	0.502	0.457	0.470	0.477	0.483	0.498
	*vf* = 0.10	0.592	0.613	0.622	0.630	0.650	0.587	0.608	0.618	0.627	0.647	0.583	0.603	0.612	0.620	0.642	0.572	0.592	0.600	0.608	0.628
	*vf* = 0.15	0.713	0.738	0.750	0.760	0.787	0.707	0.732	0.742	0.752	0.778	0.693	0.718	0.728	0.738	0.765	0.677	0.698	0.708	0.718	0.742
	*vf* = 0.20	0.825	0.855	0.867	0.880	0.910	0.813	0.843	0.855	0.867	0.895	0.797	0.825	0.835	0.847	0.875	0.772	0.797	0.807	0.817	0.842
	*vf* = 0.25	0.930	0.962	0.975	0.988	1.023	0.917	0.945	0.957	0.970	1.002	0.892	0.922	0.933	0.945	0.972	0.862	0.885	0.897	0.905	0.930
	*vf* = 0.30	1.028	1.062	1.075	1.088	1.123	1.008	1.040	1.052	1.065	1.098	0.982	1.010	1.022	1.033	1.063	0.943	0.970	0.980	0.990	1.013

**Table 3:** Informed sequential pooling, pool size *s* = 12. Values of the 1st, 25th, 50th (median), 75th, and 99th percentiles of *T/N* obtained in the set of 5,000 simulations, for all combinations of α and β in {0.5, 0.6, 0.7, 0.8}, for some values of the virus frequency (*vf* = 0.01, 0.10, 0.15, 0.20, 0.25, 0.30).

		β = 0.5					β = 0.6					β = 0.7					β = 0.8				
		1st	25th	50th	75th	99th	1st	25th	50th	75th	99th	1st	25th	50th	75th	99th	1st	25th	50th	75th	99th
α = 0.5	*vf* = 0.01	0.133	0.153	0.173	0.173	0.213	0.133	0.153	0.173	0.173	0.213	0.133	0.153	0.173	0.173	0.193	0.132	0.153	0.173	0.173	0.213
	*vf* = 0.05	0.395	0.442	0.465	0.487	0.547	0.382	0.442	0.463	0.485	0.547	0.382	0.440	0.462	0.483	0.543	0.375	0.422	0.443	0.477	0.523
	*vf* = 0.10	0.642	0.720	0.745	0.782	0.850	0.640	0.715	0.742	0.767	0.848	0.632	0.698	0.722	0.748	0.823	0.598	0.672	0.695	0.718	0.782
	*vf* = 0.15	0.828	0.898	0.935	0.958	1.038	0.828	0.893	0.932	0.957	1.022	0.805	0.872	0.897	0.933	0.997	0.763	0.828	0.852	0.888	0.953
	*vf* = 0.20	0.955	1.020	1.042	1.080	1.127	0.940	1.017	1.040	1.062	1.125	0.917	0.980	1.017	1.040	1.102	0.873	0.938	0.975	0.997	1.062
	*vf* = 0.25	1.038	1.085	1.105	1.125	1.167	1.022	1.083	1.103	1.125	1.167	0.998	1.060	1.082	1.103	1.147	0.960	1.020	1.043	1.080	1.125
	*vf* = 0.30	1.082	1.125	1.145	1.167	1.167	1.082	1.123	1.145	1.147	1.167	1.060	1.103	1.125	1.145	1.167	1.020	1.082	1.103	1.123	1.167
α = 0.6	*vf* = 0.01	0.133	0.153	0.173	0.173	0.193	0.132	0.153	0.173	0.173	0.213	0.132	0.153	0.173	0.173	0.193	0.132	0.153	0.172	0.173	0.193
	*vf* = 0.05	0.385	0.442	0.463	0.487	0.547	0.378	0.440	0.462	0.483	0.545	0.375	0.435	0.457	0.478	0.523	0.368	0.415	0.437	0.457	0.500
	*vf* = 0.10	0.640	0.715	0.742	0.767	0.847	0.635	0.698	0.723	0.760	0.825	0.612	0.675	0.698	0.722	0.785	0.568	0.632	0.653	0.675	0.737
	*vf* = 0.15	0.815	0.893	0.932	0.957	1.022	0.805	0.872	0.897	0.933	0.998	0.763	0.830	0.867	0.890	0.953	0.718	0.782	0.805	0.827	0.890
	*vf* = 0.20	0.940	1.017	1.040	1.062	1.123	0.917	0.980	1.017	1.040	1.102	0.873	0.938	0.975	0.997	1.060	0.828	0.892	0.915	0.952	0.998
	*vf* = 0.25	1.020	1.083	1.103	1.125	1.167	0.998	1.060	1.082	1.103	1.147	0.957	1.020	1.042	1.063	1.125	0.913	0.977	0.998	1.022	1.082
	*vf* = 0.30	1.080	1.123	1.145	1.147	1.167	1.058	1.103	1.123	1.145	1.167	1.020	1.082	1.102	1.123	1.167	0.980	1.040	1.062	1.083	1.145
α = 0.7	*vf* = 0.01	0.133	0.153	0.173	0.173	0.213	0.132	0.153	0.173	0.173	0.213	0.132	0.153	0.173	0.173	0.193	0.132	0.153	0.172	0.173	0.193
	*vf* = 0.05	0.380	0.440	0.462	0.483	0.543	0.377	0.437	0.458	0.478	0.525	0.372	0.417	0.437	0.458	0.518	0.350	0.393	0.413	0.435	0.477
	*vf* = 0.10	0.633	0.698	0.723	0.760	0.823	0.612	0.677	0.698	0.722	0.785	0.572	0.633	0.657	0.678	0.738	0.527	0.588	0.608	0.630	0.690
	*vf* = 0.15	0.805	0.872	0.895	0.932	0.997	0.763	0.830	0.867	0.890	0.953	0.720	0.783	0.805	0.827	0.890	0.658	0.718	0.740	0.762	0.822
	*vf* = 0.20	0.915	0.980	1.017	1.038	1.085	0.887	0.938	0.973	0.995	1.043	0.827	0.890	0.912	0.933	0.997	0.763	0.825	0.848	0.870	0.933
	*vf* = 0.25	0.998	1.060	1.082	1.103	1.147	0.957	1.018	1.040	1.062	1.107	0.912	0.975	0.997	1.018	1.080	0.865	0.913	0.935	0.973	1.037
	*vf* = 0.30	1.058	1.103	1.123	1.145	1.167	1.018	1.063	1.083	1.105	1.147	0.975	1.037	1.058	1.080	1.125	0.932	0.995	1.017	1.038	1.083
α = 0.8	*vf* = 0.01	0.132	0.153	0.173	0.173	0.213	0.132	0.153	0.173	0.173	0.213	0.132	0.153	0.172	0.173	0.193	0.130	0.152	0.172	0.173	0.193
	*vf* = 0.05	0.377	0.435	0.457	0.478	0.525	0.373	0.417	0.438	0.460	0.520	0.352	0.397	0.417	0.438	0.497	0.328	0.370	0.392	0.412	0.453
	*vf* = 0.10	0.612	0.677	0.698	0.722	0.783	0.575	0.635	0.668	0.693	0.738	0.545	0.605	0.627	0.647	0.692	0.483	0.523	0.543	0.563	0.605
	*vf* = 0.15	0.763	0.828	0.863	0.888	0.952	0.722	0.782	0.803	0.825	0.887	0.673	0.718	0.740	0.760	0.820	0.608	0.652	0.672	0.693	0.737
	*vf* = 0.20	0.872	0.933	0.955	0.978	1.040	0.823	0.887	0.908	0.930	0.975	0.762	0.810	0.842	0.865	0.910	0.695	0.740	0.762	0.783	0.842
	*vf* = 0.25	0.953	1.015	1.037	1.058	1.103	0.907	0.953	0.975	0.997	1.058	0.845	0.892	0.913	0.937	0.997	0.780	0.827	0.850	0.885	0.932
	*vf* = 0.30	1.015	1.060	1.082	1.102	1.145	0.953	1.017	1.038	1.058	1.103	0.910	0.957	0.978	1.002	1.060	0.847	0.908	0.930	0.952	1.015

**Table 4:** Informed sequential pooling, pool size *s* = 24. Values of the 1st, 25th, 50th (median), 75th, and 99th percentiles of *T/N* obtained in the set of 5,000 simulations, for all combinations of α and β in {0.5, 0.6, 0.7, 0.8}, for some values of the virus frequency (*vf* = 0.01, 0.10, 0.15, 0.20, 0.25, 0.30).

		β = 0.5					β = 0.6					β = 0.7					β = 0.8				
		1st	25th	50th	75th	99th	1st	25th	50th	75th	99th	1st	25th	50th	75th	99th	1st	25th	50th	75th	99th
α = 0.5	*vf* = 0.01	0,130	0,172	0,210	0,212	0,252	0,130	0,172	0,210	0,212	0,292	0,130	0,172	0,210	0,212	0,252	0,130	0,172	0,210	0,212	0,252
	*vf* = 0.05	0,545	0,630	0,672	0,712	0,797	0,508	0,630	0,670	0,712	0,797	0,508	0,625	0,633	0,673	0,793	0,503	0,587	0,628	0,668	0,752
	*vf* = 0.10	0,835	0,918	0,960	1,000	1,083	0,798	0,918	0,958	1,000	1,043	0,795	0,878	0,918	0,960	1,043	0,753	0,838	0,878	0,918	1,003
	*vf* = 0.15	0,960	1,042	1,043	1,083	1,083	0,958	1,042	1,043	1,083	1,083	0,920	1,002	1,042	1,043	1,083	0,917	1,000	1,002	1,042	1,083
	*vf* = 0.20	1,003	1,083	1,083	1,083	1,083	1,002	1,083	1,083	1,083	1,083	1,000	1,042	1,083	1,083	1,083	0,960	1,042	1,083	1,083	1,083
	*vf* = 0.25	1,042	1,083	1,083	1,083	1,083	1,042	1,083	1,083	1,083	1,083	1,042	1,083	1,083	1,083	1,083	1,042	1,083	1,083	1,083	1,083
	*vf* = 0.30	1,083	1,083	1,083	1,083	1,083	1,043	1,083	1,083	1,083	1,083	1,042	1,083	1,083	1,083	1,083	1,042	1,083	1,083	1,083	1,083
α = 0.6	*vf* = 0.01	0,130	0,172	0,210	0,212	0,292	0,130	0,172	0,210	0,212	0,252	0,128	0,170	0,210	0,212	0,252	0,128	0,170	0,208	0,210	0,252
	*vf* = 0.05	0,543	0,630	0,670	0,712	0,797	0,507	0,625	0,635	0,673	0,793	0,503	0,588	0,628	0,668	0,752	0,463	0,545	0,587	0,627	0,710
	*vf* = 0.10	0,798	0,918	0,958	1,000	1,043	0,795	0,878	0,920	0,960	1,043	0,753	0,838	0,878	0,920	1,002	0,710	0,795	0,835	0,877	0,960
	*vf* = 0.15	0,958	1,003	1,043	1,083	1,083	0,918	1,002	1,042	1,043	1,083	0,917	1,000	1,002	1,042	1,083	0,837	0,958	1,000	1,002	1,083
	*vf* = 0.20	1,002	1,083	1,083	1,083	1,083	1,000	1,042	1,083	1,083	1,083	0,958	1,042	1,043	1,083	1,083	0,958	1,000	1,042	1,083	1,083
	*vf* = 0.25	1,042	1,083	1,083	1,083	1,083	1,042	1,083	1,083	1,083	1,083	1,000	1,083	1,083	1,083	1,083	1,000	1,042	1,083	1,083	1,083
	*vf* = 0.30	1,043	1,083	1,083	1,083	1,083	1,042	1,083	1,083	1,083	1,083	1,042	1,083	1,083	1,083	1,083	1,042	1,083	1,083	1,083	1,083
α = 0.7	*vf* = 0.01	0,130	0,172	0,210	0,212	0,292	0,130	0,172	0,210	0,212	0,252	0,130	0,170	0,210	0,212	0,252	0,128	0,170	0,210	0,210	0,252
	*vf* = 0.05	0,507	0,593	0,635	0,673	0,793	0,503	0,588	0,628	0,670	0,752	0,465	0,547	0,587	0,627	0,708	0,422	0,503	0,543	0,583	0,627
	*vf* = 0.10	0,793	0,878	0,918	0,960	1,042	0,752	0,837	0,877	0,918	1,002	0,710	0,793	0,835	0,877	0,960	0,667	0,750	0,792	0,833	0,917
	*vf* = 0.15	0,918	1,000	1,042	1,043	1,083	0,877	0,960	1,000	1,042	1,083	0,835	0,918	0,958	1,000	1,083	0,792	0,875	0,917	0,958	1,042
	*vf* = 0.20	1,000	1,042	1,083	1,083	1,083	0,958	1,042	1,042	1,083	1,083	0,917	1,000	1,042	1,042	1,083	0,875	0,958	1,000	1,042	1,083
	*vf* = 0.25	1,042	1,083	1,083	1,083	1,083	1,000	1,042	1,083	1,083	1,083	1,000	1,042	1,083	1,083	1,083	0,958	1,042	1,042	1,083	1,083
	*vf* = 0.30	1,042	1,083	1,083	1,083	1,083	1,042	1,083	1,083	1,083	1,083	1,000	1,083	1,083	1,083	1,083	1,000	1,042	1,083	1,083	1,083
α = 0.8	*vf* = 0.01	0,128	0,170	0,210	0,212	0,252	0,128	0,170	0,210	0,212	0,252	0,128	0,170	0,208	0,210	0,252	0,127	0,168	0,170	0,210	0,252
	*vf* = 0.05	0,503	0,587	0,628	0,668	0,752	0,463	0,547	0,587	0,628	0,710	0,425	0,503	0,543	0,583	0,627	0,380	0,460	0,500	0,502	0,542
	*vf* = 0.10	0,753	0,835	0,877	0,917	1,000	0,708	0,792	0,833	0,837	0,918	0,627	0,710	0,752	0,792	0,875	0,583	0,667	0,708	0,710	0,792
	*vf* = 0.15	0,877	0,958	1,000	1,002	1,083	0,833	0,917	0,958	0,960	1,042	0,753	0,875	0,877	0,917	1,000	0,708	0,792	0,833	0,875	0,958
	*vf* = 0.20	0,958	1,002	1,042	1,083	1,083	0,917	1,000	1,000	1,042	1,083	0,875	0,918	0,958	1,000	1,083	0,792	0,875	0,917	0,958	1,042
	*vf* = 0.25	1,000	1,042	1,083	1,083	1,083	0,958	1,042	1,042	1,083	1,083	0,917	1,000	1,042	1,042	1,083	0,875	0,958	1,000	1,042	1,083
	*vf* = 0.30	1,042	1,083	1,083	1,083	1,083	1,000	1,042	1,083	1,083	1,083	0,958	1,042	1,042	1,083	1,083	0,917	1,000	1,042	1,042	1,083

As above mentioned, we started with α and β equal to 0.5, because this is substantially equivalent to uninformative prior criteria. As α and/or β increase, we observe that the number of required tests decreases, and this decrease is larger when the virus frequency is greater. When *vf* is below 5%, random pooling and informed pooling are almost equivalent. With a low *vf*, sequential random pooling was, however, already very performant, substantially decreasing the number of tests with respect to separate individual tests. For larger *vf*, the curves corresponding to random assignment and informed pooling separate more and more. This implies that reliable informed pooling increases the performance of the pooling exactly when the situation is less favourable. For example, with a pool size equal to 12, with a random assignment, the median of *T/N* is equal to 1 when *vf* ≈ 0.18 (making random pooling application questionable). Conversely, if informed pooling is performed with α = β = 0.8, at the same *vf*, the median of *T/N* is approximately equal to 0.73. With α = β = 0.8, pooling is still efficient (*T/N<1)* even if the virus frequency approaches 30%. In summary, reliable informed pooling makes the performance path much more linear than we observed for random pooling, even if we use larger pools. That is, larger pools, besides providing substantial savings for low *vf*, ensure efficiency even for larger *vf* if epidemiological criteria provide reliable information.

## Discussion

Every successful strategy for controlling the SARS-CoV-2 infection depends on timely diagnosis. Hence, there is an urgent need for systematic population screening on a massive scale. Currently, around the world, there is a plethora of different scenarios depending on the spread of infection. Transmission of the SARS-CoV-2 has a high degree of heterogeneity across diverse environments, and even within a single country, there are categories with different contagion risks; for each category, the optimal monitoring frequency must be determined to prevent outbreaks. Moreover, in this variegated context, there are completely different economic situations, and the pooling strategy can become truly attractive for countries with fewer resources. The study published by [10] is certainly an excellent starting point for the evaluation of an alternative approach to individual analysis of swab samples for the RT-PRC based diagnosis of the SARS-CoV-2, but some additional considerations are needed.

First, it must be highlighted that in the study [10], 292 pools of 9 or 10 samples were created and two positive cases in a collection of 2888 samples were found. The one-step pooling method gave excellent results because the frequency of the virus in the analyzed samples was extremely low (0.07%). Second, if it were possible to roughly estimate the frequency of the virus in the collection as being lower than 5%, our data suggest increasing the pool size. Using a pool size of 24, for example, the screening of the 2888 samples would need about 120 tests instead of 292.

The most difficult samples to be detected are those from patients who are in the early or late stage of infection, because of the lower viral load [14]. Furthermore, these samples risk going undetected as false negatives if the pooling procedure causes dilution or fractionation of the positive specimen. There are ongoing studies attempting to determine the ideal protocol and possible technical solutions to reduce this risk ([21–23]). However, it should also be stressed that the pooling method is so efficient that it could also allow for an increase in the frequency of serial testing and a timed monitoring—an effective strategy especially for subjects with a higher risk of infection.

Our most straightforward result is that the sequential pooling approach is more efficient than the one-step pooling method. In addition, the informed version of sequential pooling can further improve its performance, in particular for larger size pools and moderate to large virus frequency. Table 5 broadly describes practical suggestions to decide the pool size, *s*, according to rough assumptions about the virus frequency, both for random and informed sequential pooling. Larger pools ensure a significant reduction in the number of tests when *vf* is small. Smaller pools may be a conservative approach when dealing with cohorts with heavier exposure. Finally, indications are also given to avoid the use of pooling when virus frequency is higher and random pooling would result in a waste of resources, since too many pools are expected to yield a positive result.

**Table 5:** Summary of practical indications for pooling creation.

Random sequential pooling	Informed sequential pooling (α,β≥0.7)
If we can assume a *vf* below 10%, pools with sizes ranging from 10 to 15 can provide relevant savings in the number of tests. With *vf* below 5%, even stronger savings can be obtained with pool sizes increased to 20 or 25.	If we can assume a *vf* below 10%, very large pools, with sizes ranging from 20 to 25 could substantially reduce the number of tests. Pools with sizes equal to 30 or 40 are a good strategy with *vf* below 5%.
For situations in which *vf* may reach 10%-20% of the cohort, there can still be a moderate reduction in the number of tests, with pools of size between 5 and 8.	For situations in which *vf* may reach 10%-20% of the cohort, we can still have a relevant reduction in the number of tests, with pools of size between 12 and 20.
For situations in which there is the risk of a *vf* value above 20% of the cohort, pooling strategies should be avoided.	For situations when there is the risk of a *vf* value above 20% of the cohort, a moderate reduction can be attained with pool sizes about 12, to be further reduced to 8 if the *vf* may exceed 25%.

## Supporting information

S1 Fig Random sequential pooling. The plotted curves represent the 1st, 25th, 50th (median), 75th, and 99th percentiles of *T/N* obtained in the set of 5,000 simulations, for a three values of the pool size *s* from 2 to 150.
(TIF)

S2 Fig Informed sequential pooling. The plotted curves represent the 50th percentile (median) of *T/N* obtained in the set of 5,000 simulations, for values of the pool size *s* ranging from 2 to 50. The plots were obtained with α = 0.5 and α = 0.6, combined with β = 0.5, 0.6, 0.7, 0.8.
(TIF)

S3 Fig Informed sequential pooling. The plotted curves represent the 50th percentile (median) of *T/N* obtained in the set of 5,000 simulations, for values of the pool size *s* ranging from 2 to 50. The plots were obtained with α = 0.7 and α = 0.8, combined with β = 0.5, 0.6, 0.7, 0.8.
(TIF)

## References

[1] Panati K, Tatireddygari VRA, Narala VR. An overview on COVID-19 pandemic: from discovery to treatment. Infect Disord Drug Targets. 2020 11 8 10.2174/1871526520666201109115820 Epub ahead of print. . 33167846

[2] Mondelli MU, Colaneri M, Seminari EM, Baldanti F, Bruno R. Low risk of SARS-CoV-2 transmission by fomites in real-life conditions. Lancet Infect Dis. 2020 9 29:S1473-3099(20)30678-2. 10.1016/S1473-3099(20)30678-2 Epub ahead of print. 33007224 PMC7524520

[3] Al-Tawfiq JA. Asymptomatic coronavirus infection: MERS-CoV and SARS-CoV-2 (COVID-19). Travel Med Infect Dis. 2020; 27:101608 10.1016/j.tmaid.2020.101608 32114075 PMC7102602

[4] Wang D, Hu B, Hu C, Zhu F, Liu X, Zhang J, et al Clinical characteristics of 138 hospitalized patients with 2019 novel coronavirus–infected pneumonia in Wuhan, China. JAMA. 2020 10.1001/jama.2020.1585 32031570 PMC7042881

[5] Lan L, Xu D, Ye G, Xia C, Wang S, Li Y, et al Positive RT-PCR Test Results in Patients Recovered From COVID-19. JAMA. 2020 2 27 10.1001/jama.2020.2783 [Epub ahead of print] 32105304 PMC7047852

[6] Charlton CL, Babady E, Ginocchio CC, Hatchette TF, Jerris RC, Li Y, et al Practical guidance for clinical microbiology laboratories: viruses causing acute respiratory tract infections. Clin Microbiol Rev. 2019;32(1). 10.1128/CMR.00042-18 30541871 PMC6302358

[7] Azzi L, Carcano G, Gianfagna F, Grossi P, Gasperina DD, Genoni A, et al University of Insubria COVID-19 Task Force, Baj A5. SALIVA IS A RELIABLE TOOL TO DETECT SARS-CoV-2. J Infect. 2020: S0163-4453(20)30213-9. 10.1016/j.jinf.2020.04.005 [Epub ahead of print] 32298676 PMC7194805

[8] Falsey AR, Formica MA, Walsh EE. Simple method for combining sputum and nasal samples for virus detection by reverse transcriptase PCR. J Clin Microbiol. 2012; 50(8):2835 10.1128/JCM.01473-12 22692748 PMC3421505

[9] Ott IM, Strine MS, Watkins AE, Boot M, Kalinich CC, Harden CA, et al Simply saliva: stability of SARS-CoV-2 detection negates the need for expensive collection devices. medRxiv [Preprint]. 2020 8 4:2020.08.03.20165233. 10.1101/2020.08.03.20165233 32793924 PMC7418742

[10] Hogan CA, Sahoo MK, Pinsky BA. Sample Pooling as a Strategy to Detect Community Transmission of SARS-CoV-2. JAMA. 2020 10.1001/jama.2020.5445 32250394 PMC7136853

[11] Dorfman R. The Detection of Defective Members of Large Populations. Ann Math Statist. 1943; 14(4):436–440. 10.1214/aoms/1177731363

[12] Yelin I, Aharony N, Shaer Tamar E, Argoetti A, Messer E, Berenbaum D, et al Evaluation of COVID-19 RT-qPCR test in multi-sample pools. Clin Infect Dis. 2020 5 2:ciaa531 10.1093/cid/ciaa531 32358960 PMC7197588

[13] Gan Yichuan, Du Lingyan, Faleti Oluwasijibomi Damola Jing Huang, Xiao Gang, Lyu Xiaoming. Sample Pooling as a Strategy of SARS-COV-2 Nucleic Acid Screening Increases the False-negative Rate, medRxiv 2020.05.18.20106138; 10.1101/2020.05.18.20106138

[14] Lippi G, Simundic AM, Plebani M. Potential preanalytical and analytical vulnerabilities in the laboratory diagnosis of coronavirus disease 2019 (COVID-19). Clin Chem Lab Med. 2020;58(7):1070–1076. 10.1515/cclm-2020-0285 32172228

[15] Watkins AE, Fenichel EP, Weinberger DM, Vogels CBF, Brackney DE, Casanovas-Massana A, et al Pooling saliva to increase SARS-CoV-2 testing capacity. medRxiv [Preprint]. 2020 9 3:2020.09.02.20183830. 10.1101/2020.09.02.20183830 32909003 PMC7480055

[16] Larremore DB, Wilder B, Lester E, Shehata S, Burke JM, Hay JA, et al Test sensitivity is secondary to frequency and turnaround time for COVID-19 surveillance. medRxiv 2020.06.22.20136309; 10.1101/2020.06.22.20136309 33219112 PMC7775777

[17] Sasmita PA, Sha M, Yu-Ju W, Yu-Ping M, Rui-Xue Y, Qing-Zhi W, et al Epidemiology, causes, clinical manifestation and diagnosis, prevention and control of coronavirus disease (COVID-19) during the early outbreak period: a scoping review. Infect Dis Poverty. 2020; 9: 29 10.1186/s40249-020-00646-x 32183901 PMC7079521

[18] Fehr AR, Channappanavar R, Perlman S. Middle East respiratory syndrome: emergence of a pathogenic human coronavirus. Annu Rev Med. 2017;68:387–399. 10.1146/annurev-med-051215-031152 27576010 PMC5353356

[19] Chen N, Zhou M, Dong X, Qu J, Gong F, Han Y, et al Epidemiological and clinical characteristics of 99 cases of 2019 novel coronavirus pneumonia in Wuhan, China: a descriptive study. Lancet. 2020;395:507–513. 10.1016/S0140-6736(20)30211-7 32007143 PMC7135076

[20] Backer JA, Klinkenberg D, Wallinga J. The incubation period of 2019-nCoV infections among travellers from Wuhan. China Euro Surveill. 2020; 10.2807/1560-7917.ES.2020.25.5.2000062 32046819 PMC7014672

[21] Schmidt M, Hoehl S, Berger A, Zeichhardt H, Hourfar K, Ciesek S, et al Novel multiple swab method enables high efficiency in SARS-CoV-2 screenings without loss of sensitivity for screening of a complete population. Transfusion. 2020 7 6: 10.1111/trf.15973 32627200 PMC7361511

[22] Lu Renfei, Wang Jian, Li Min, Wang Yaqi, Dong Jia, Cai Weihua. SARS-CoV-2 detection using digital PCR for COVID-19 diagnosis, treatment monitoring and criteria for discharge, medRxiv. 10.1101/2020.03.24.20042689

[23] Wacharapluesadee S, Kaewpom T, Ampoot W, Siriporn G, Worrawat K, Kanthita W et al Evaluating the efficiency of specimen pooling for PCR-based detection of COVID-19 [published online ahead of print, 2020 May 13]. J Med Virol. 2020; 10.1002/jmv.26005 32401343 PMC7272832

