# Supplementary figures and images for: Informed sequential pooling approach to detect SARS-CoV-2 infection
Source: PLoS One. 2020 Dec 30;15(12):e0244475. doi: 10.1371/journal.pone.0244475 (PMC7773195; doi:10.1371/journal.pone.0244475)

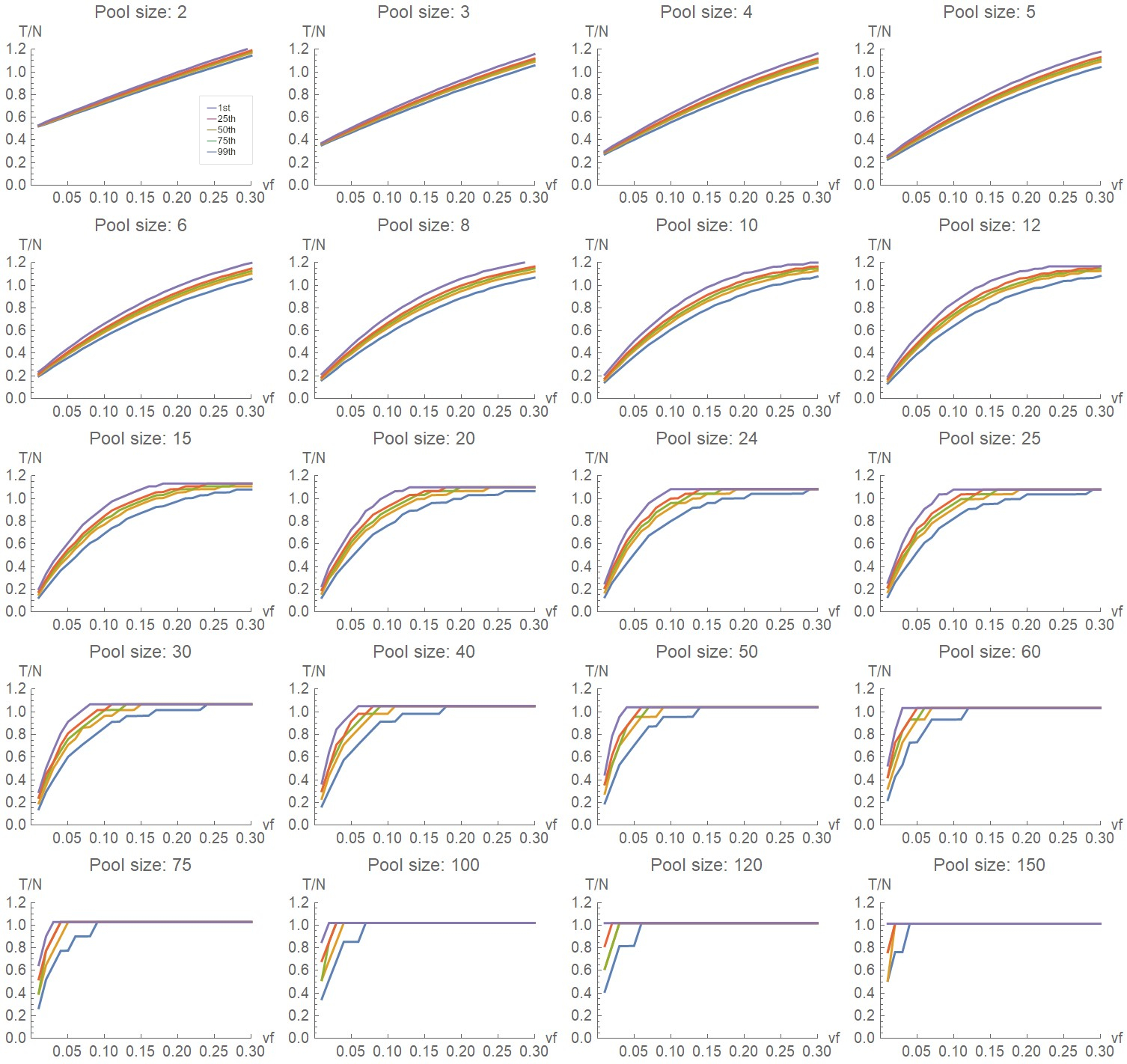

Supplement: S1 Fig — The plotted curves represent the 1st, 25th, 50th (median), 75th, and 99th percentiles of T/N obtained in the set of 5,000 simulations, for a three values of the pool size s from 2 to 150. (TIF) [file pone.0244475.s001.tif]

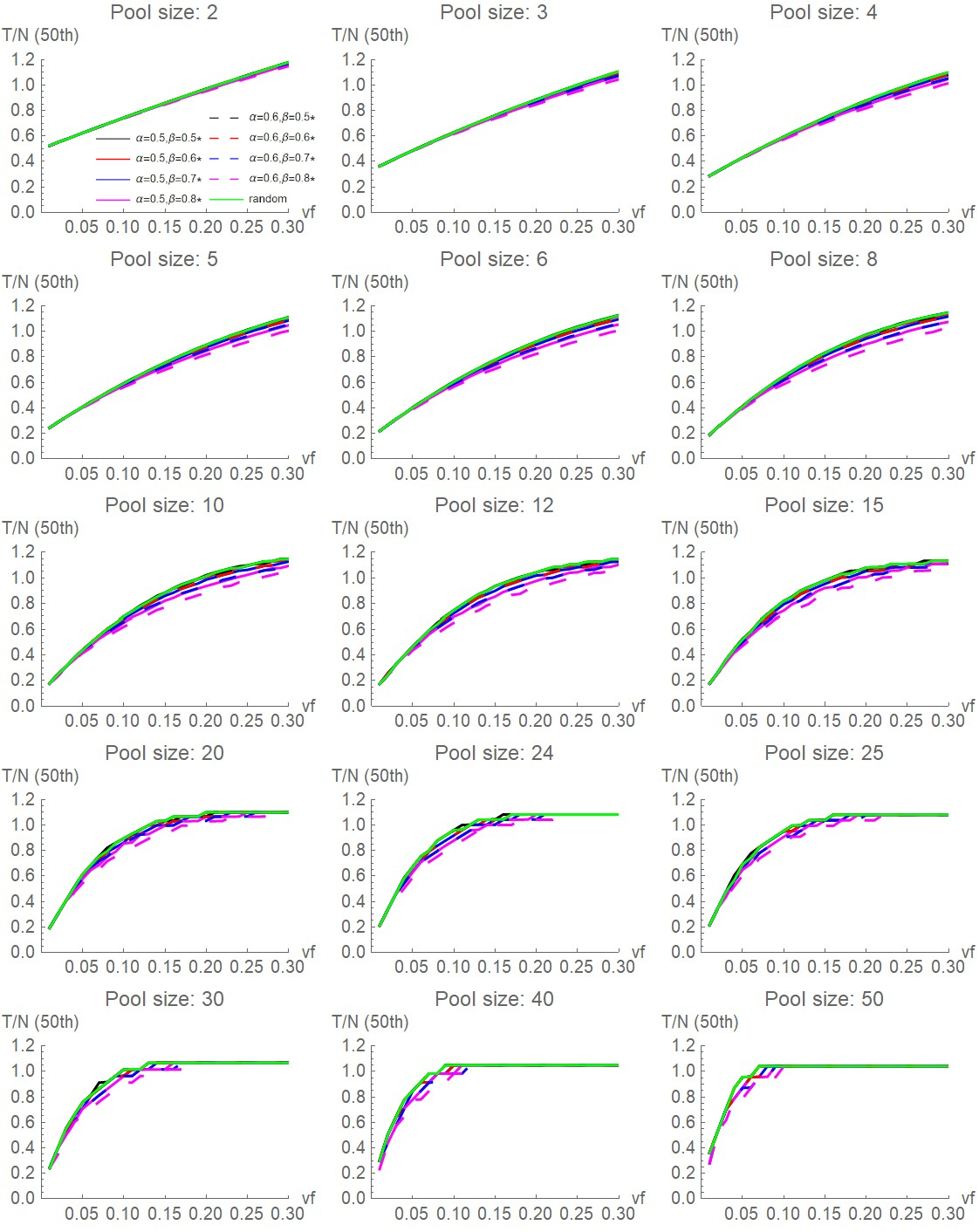

Supplement: S2 Fig — The plotted curves represent the 50th percentile (median) of T/N obtained in the set of 5,000 simulations, for values of the pool size s ranging from 2 to 50. The plots were obtained with α = 0.5 and α = 0.6, combined with β = 0.5, 0.6, 0.7, 0.8. (TIF) [file pone.0244475.s002.tif]

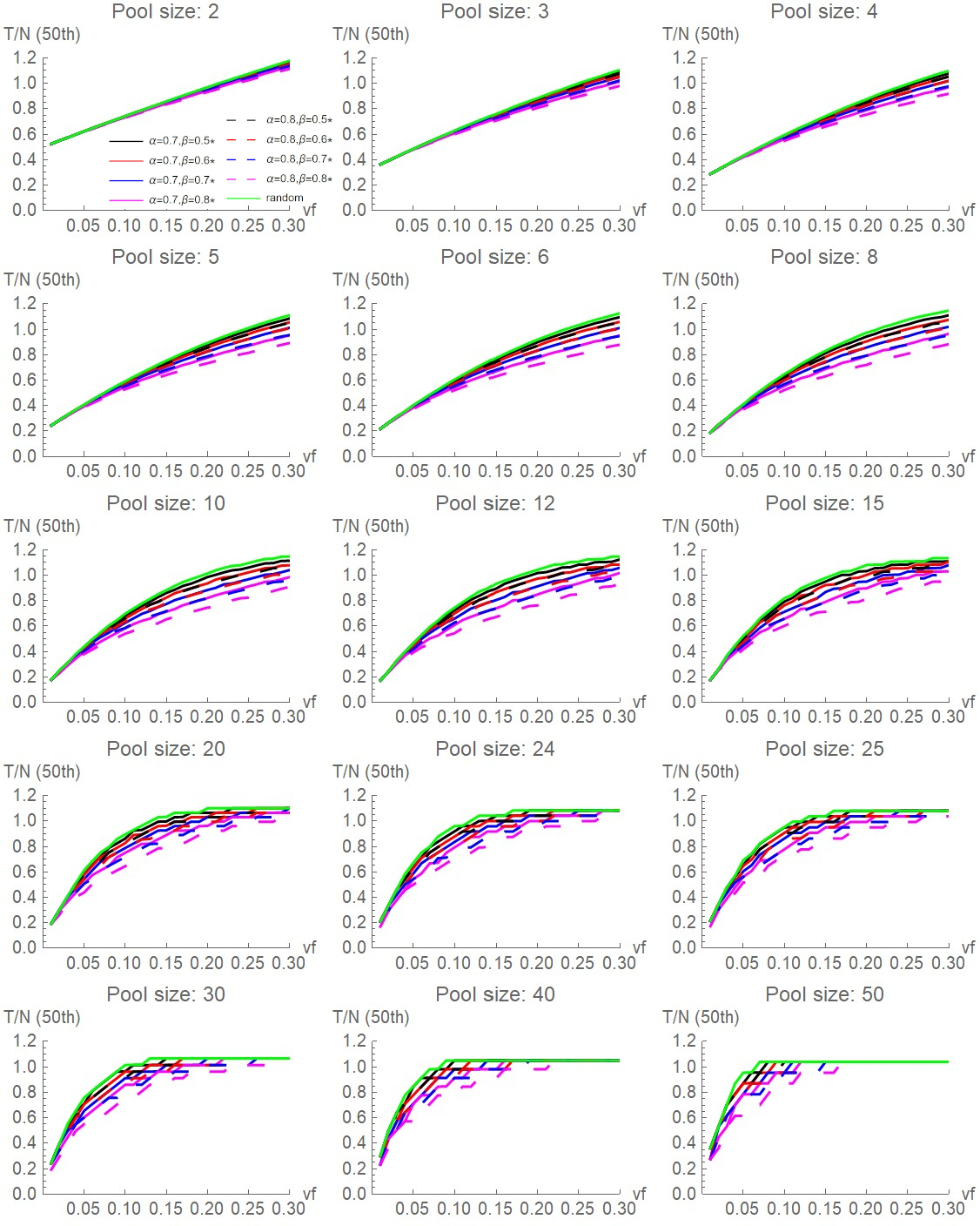

Supplement: S3 Fig — The plotted curves represent the 50th percentile (median) of T/N obtained in the set of 5,000 simulations, for values of the pool size s ranging from 2 to 50. The plots were obtained with α = 0.7 and α = 0.8, combined with β = 0.5, 0.6, 0.7, 0.8. (TIF) [file pone.0244475.s003.tif]
